# Comparison between experimental and satellite temperature datasets in Covenant University

**DOI:** 10.1016/j.dib.2018.08.012

**Published:** 2018-08-09

**Authors:** Sayo A. Akinwumi, Temidayo V. Omotosho, Mojisola R. Usikalu, Oluwole A. Odetunmibi, Oluwafunmilayo O. Ometan, Mustapha O. Adewusi, Maxwell Omeje, Emmanuel S. Joel

**Affiliations:** a Department of Physics, Covenant University, Ota, Nigeria; b Department of Mathematics, Covenant University, Ota, Nigeria; c Department of Physics, Lagos state University, Ojo, Lagos state, Nigeria

**Keywords:** Temperature, Satellite communication, Clear-sky attenuation, Electromagnetic wave

## Abstract

This article contains the ground and satellite meteorological data sets of clear-sky temperature events for five years (2012–2016) in Covenant University, Ota, Ogun State, Nigeria. The satellite data were obtained from Atmospheric Infrared Sounder (AIRS) while the ground data information were acquired from Davis weather station data logger-vantage pro2. These data were acquired from propagation study that used same location (Lat: 6.67°N and Long: 3.23°E) for both satellite data and radiometer directed along the same path by providing information about the temperature. The data sets were assessed and evaluated by means of a descriptive statistics. There was perfect agreement between the two data. The peak temperature events occurs between the months of November and April for the five years of observation for both Ota and AIRS Satellite. The data from this article can be used for further studies on non-rainy attenuation effect in the study area.

**Specifications Table**

**Table:** 

Subject area	*Communication and Atmospheric physics*
More specific subject area	*Radiowave propagation*
Type of data	*Table and figure*
How data was acquired	*Both primary and Secondary data*
Data format	*Raw and analyzed*
Experimental factors	*Atmospheric Infrared Sounder (AIRS) and Davis weather station data logger-vantage pro2*
Experimental features	*Computational Analysis: Descriptive statistics*
Data source location	*Covenant University, Ota, Ogun State, Nigeria (Lat: 6.67*^*o*^*N and Long: 3.23*^*o*^*E)*
Data accessibility	*All the data are available in this article*

**Value of the data**

• The data could be used to estimate clear-air attenuation in troposphere.
• The technique employed here can be replicated on temperature across Nigeria.
• The information provided in this data can be used for purpose of citing antenna during rainy season and non-rainy season.
• This data may be appreciated in understanding tropospheric scintillation and gas attenuation.

## 1 Ground and satellite temperature data

*Both the ground measured data and satellite data for this article were collected from Davis weather station data logger-vantage pro2 located at the top roof of College of Science and Technology of Covenant University, Nigeria and Atmospheric Infrared Sounder (AIRS) for five years between January 2012 and December 2016 respectively. The data major input parameter is temperature as presented in* Table 1, Table 2, Table 3, Table 4, Table 5*. The ground temperature data gathered were arranged on daily average data that is based on one-minute data and consequently used to achieve the monthly data. The monthly averages of both experimental and satellite data five years is a good description of the seasonal performance of temperature as shown in* Table 1, Table 2, Table 3, Table 4, Table 5*. The statistical descriptive analyses were additional carried out on the data sets for more investigation.*

**Table 1:** 2012 data descriptive statistics.

Temperature (°C)	CU 2012	SAT 2012
Mean	25.8611111	26.5479950
Median	25.5700000	26.9559375
Mode	24.17000^a^	24.58438^a^
Std. Deviation	1.13467445	.89741231
Variance	1.287	.805
Skewness	.196	-1.111
Std. Error of Skewness	.717	.637
Kurtosis	-.717	.508
Std. Error of Kurtosis	1.400	1.232
Minimum	24.17000	24.58438
Maximum	27.71000	27.56094

**Table 2:** 2013 data descriptive statistics.

Temperature (°C)	CU 2013	SAT 2013
Mean	26.3866667	26.6234378
Std. Error of Mean	.37912645	.29400622
Median	26.6400000	26.9007815
Mode	24.25000^a^	24.79531^a^
Std. Deviation	1.31333256	1.01846744
Variance	1.725	1.037
Skewness	-.242	-1.007
Std. Error of Skewness	.637	.637
Kurtosis	-1.000	-.467
Std. Error of Kurtosis	1.232	1.232
Range	4.07000	2.84375
Minimum	24.25000	24.79531
Maximum	28.32000	27.63906

**Table 3:** 2014 data descriptive statistics.

Temperature (°C)	CU 2014	SAT 2014
Mean	26.4341667	26.4286462
Std. Error of Mean	.32538429	.26910302
Median	26.9150000	26.8617190
Mode	27.34000	24.57656^a^
Std. Deviation	1.12716425	.93220021
Variance	1.270	.869
Skewness	-.731	-1.185
Std. Error of Skewness	.637	.637
Kurtosis	-.956	-.116
Std. Error of Kurtosis	1.232	1.232
Range	3.19000	2.74219
Minimum	24.37000	24.57656
Maximum	27.56000	27.31875

**Table 4:** 2015 data descriptive statistics.

Temperature (°C)	CU 2015	SAT 2015
Mean	26.6541667	26.6588283
Std. Error of Mean	.36504142	.25182488
Median	26.4250000	26.7342190
Mode	25.22000^a^	25.15469^a^
Std. Deviation	1.26454058	.87234696
Variance	1.599	.761
Skewness	.001	-.351
Std. Error of Skewness	.637	.637
Kurtosis	-1.470	-.955
Std. Error of Kurtosis	1.232	1.232
Range	3.63000	2.63281
Minimum	24.79000	25.15469
Maximum	28.42000	27.78750

**Table 5:** 2016 data descriptive analysis.

Temperature (°C)	CU 2016	SAT 2016
Mean	27.0483333	26.4705210
Std. Error of Mean	.40702512	.32060799
Median	27.4550000	26.5843750
Mode	28.76000	25.18594^a^
Std. Deviation	1.40997636	.96182398
Variance	1.988	.925
Skewness	-.210	-.026
Std. Error of Skewness	.637	.717
Kurtosis	-1.559	-1.648
Std. Error of Kurtosis	1.232	1.400
Range	3.80000	2.62500
Minimum	24.96000	25.18594
Maximum	28.76000	27.81094

### 1.1 Temperature values recorded in 2012

**Fig. 1:**
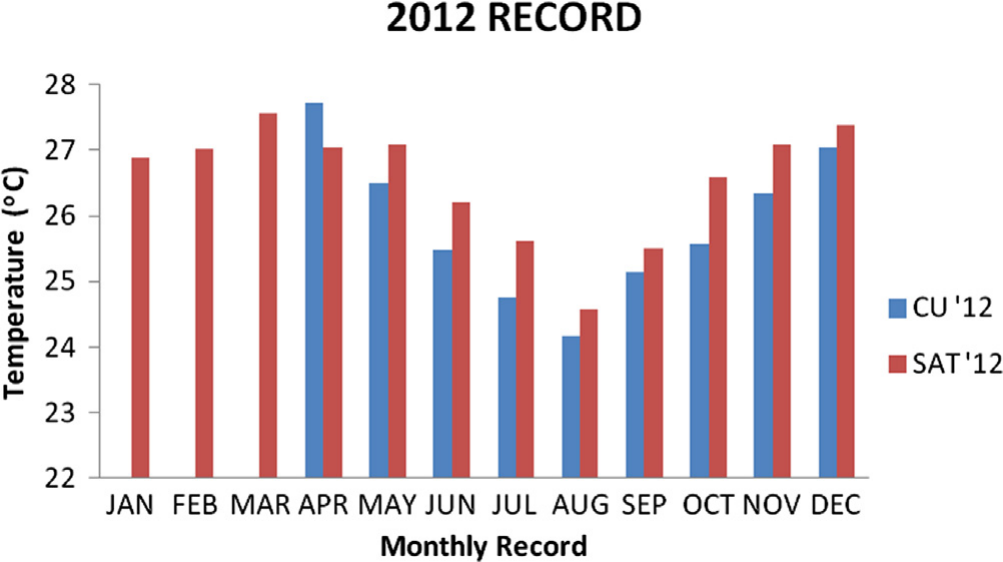
Chart for 2012 data.

Descriptive analysis was carried on the data recorded from the two locations for the year 2012 and the result is presented in the Table 1 below and the bar chart for the data is also presented in Fig. 1.

### 1.2 Temperature values recorded in 2013

**Fig. 2:**
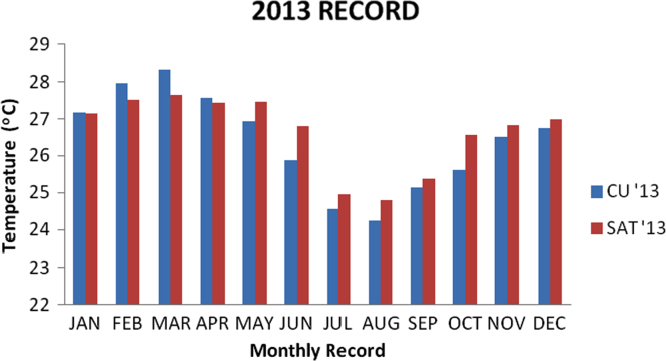
Chart for 2013 data.

Descriptive analysis was carried on the data recorded from the two locations for the year 2013 and the result is presented in the Table 2 below and the bar chart for the data is also presented in Fig. 2.

### 1.3 Temperature values recorded in 2014

**Fig. 3:**
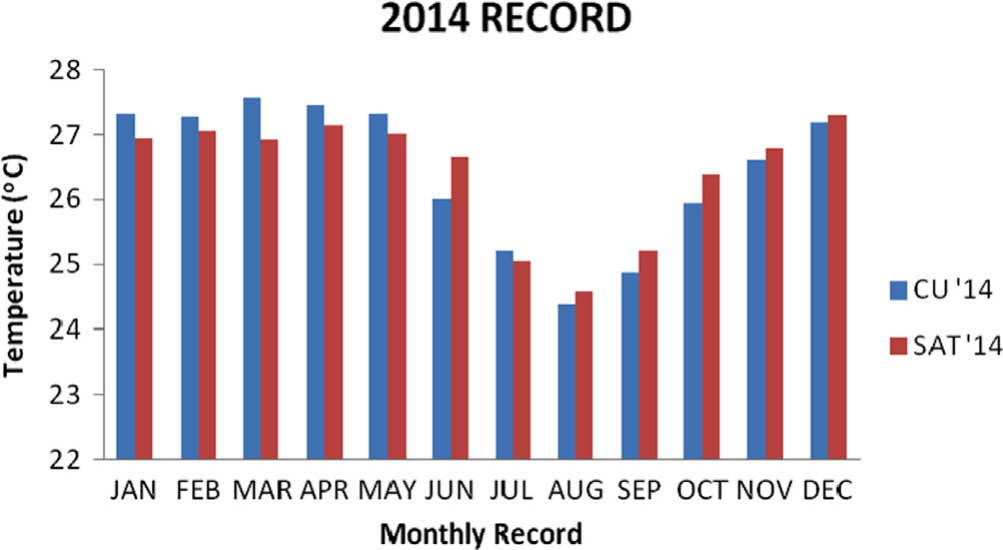
Chart for 2014 data.

The recoded data from the two locations were analyzed using descriptive analysis methods. The result is presented in the Table 3 below and the bar chart for the data is also presented in Fig. 3.

### 1.4 Temperature values recorded in 2015

**Fig. 4:**
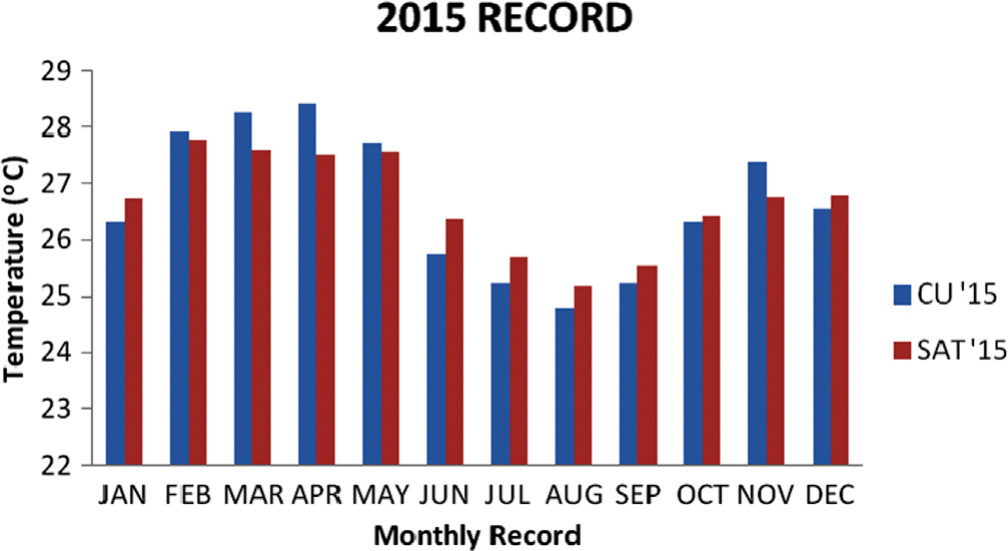
Chart for 2015 Data.

The recoded data from the two locations for the year 2015 were analyzed using descriptive analysis methods. The result is presented in the Table 4 below and the bar chart for the data is also presented in Fig. 4.

### 1.5 Temperature values recorded in 2016

**Fig. 5:**
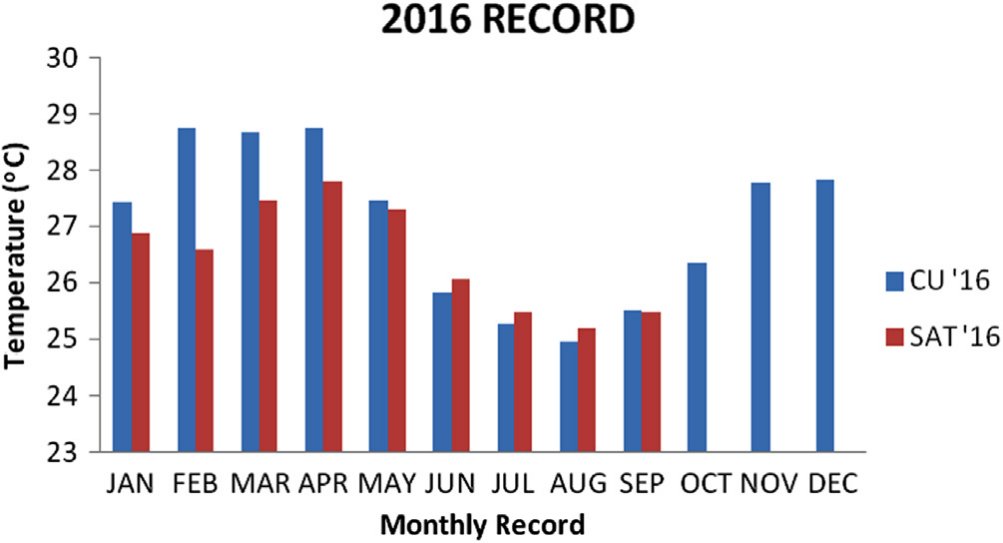
Chart for 2016 Data.

Descriptive analysis was carried on the data recorded from the two locations for the year 2016 and the result is presented in the Table 5 below and the bar chart for the data is also presented in Fig. 5.

## 2 Experimental design, materials and methods

Reasonably, many researches have been conducted on clear-sky temperature [1], [2], [3], [4], [5], [6], [7]. Similarly, statistical tools were equally used by [8], [9], [10], [11]. The data employs for this article were both the ground measured data and satellite data for the period of 5 years (2012 to 2016) Covenant University, Ota. It was gathered from Davis weather station data logger-vantage pro2 located at Covenant University, Nigeria and Atmospheric Infrared Sounder (AIRS) located in USA.
